# Using qualitative research within complex interventions: lessons from the podosa (prevention of diabetes and obesity in South Asians) trial

**DOI:** 10.1186/1745-6215-14-S1-P101

**Published:** 2013-11-29

**Authors:** Zoe Morrison, Anne Douglas, Raj Bhopal, Aziz Sheikh

**Affiliations:** 1 The University of Edinburgh, Edinburgh, UK

## Background

We conducted a qualitative study to gain insight into the high retention rate and variable outcomes achieved in the 3-year PODOSA (Prevention of Diabetes and Obesity in South Asians) family-based cluster randomised controlled trial of a dietitian-delivered lifestyle modification intervention.

## Methods

We interviewed 21 trial participants and family volunteers following trial completion. Purposive sampling ensured representation of the diversity within the trial population. Data were thematically analysed. Findings were shared with trial dietiticans once the trial had closed.

## Findings

Many participants were aware of their family history of diabetes and attracted by the availability of information and monitoring. Offering home or clinic-based interventions, communication in the participant's chosen language(s) and excellent relationships between participants and dieticians contributed to retention. Adaptations in food choices were accommodated by participants, although community and faith based considerations made consistent adherence challenging. Participants found increasing their level of physical activity difficult given other demands, including long working hours, physically demanding employment and domestic commitments.

## Interpretation

Undertaking qualitative research allowed in-depth insight into participation, retention and adherence in the PODOSA trial. The main benefit participants sought was information regarding diabetes. Home-based interventions and continuity in dieticians were included in trial design, but it was not anticipated that these relationships would contribute so greatly to retention. Adaptations in food choices were not felt to be an extra burden upon participants but were made more difficult due to cultural considerations. Increasing levels of physical activity were felt to be an additional burden, again augmented by cultural considerations.

